# Strain hardening recovery mediated by coherent precipitates in lightweight steel

**DOI:** 10.1038/s41598-021-93795-4

**Published:** 2021-07-14

**Authors:** Sung-Dae Kim, Seong-Jun Park, Jae hoon Jang, Joonoh Moon, Heon-Young Ha, Chang-Hoon Lee, Hyungkwon Park, Jong-Ho Shin, Tae-Ho Lee

**Affiliations:** 1 Advanced Metals Division, Korea Institute of Materials Science, 797 Changwondaero, Changwon, 51508 Republic of Korea; 2 Materials and Manufacturing Technology Development Center, Corporate Research and Development Institute, Doosan Heavy Industries and Construction Co. LTD, 22 Doosanvolvo-ro, Changwon, Gyeongnam 51711 Republic of Korea

**Keywords:** Metals and alloys, Mechanical properties

## Abstract

We investigated the effect of κ-carbide precipitates on the strain hardening behavior of aged Fe–Mn-Al-C alloys by microstructure analysis. The κ-carbides-strengthened Fe–Mn-Al-C alloys exhibited a superior strength-ductility balance enabled by the recovery of the strain hardening rate. To understand the relation between the κ-carbides and strain hardening recovery, dislocation gliding in the aged alloys during plastic deformation was analyzed through in situ tensile transmission electron microscopy (TEM). The in﻿ situ TEM results confirmed the particle shearing mechanism leads to planar dislocation gliding. During deformation of the 100 h-aged alloy, some gliding dislocations were strongly pinned by the large κ-carbide blocks and were prone to cross-slip, leading to the activation of multiple slip systems. The abrupt decline in the dislocation mean free path was attributed to the activation of multiple slip systems, resulting in the rapid saturation of the strain hardening recovery. It is concluded that the planar dislocation glide and sequential activation of slip systems are key to induce strain hardening recovery in polycrystalline metals. Thus, if a microstructure is designed such that dislocations glide in a planar manner, the strain hardening recovery could be utilized to obtain enhanced mechanical properties of the material.

## Introduction

A class of high-Mn steels provides an alternative way to increase their specific strength by reducing their specific weight, leading them to be known as “lightweight steels”^1–4^. Lightweight steels are realized by adding the characteristic light element, i.e., Al, where 1 wt. % reduces the specific weight of steels by approximately 1.5 wt.%^2–4^. This alloy system is characterized by a high content of Mn (18–30 wt.%) and Al (< 12 wt.%) along with C (0.6–1.8 wt.%)^1–18^. In particular, the addition of Al to the alloys not only promotes weight reduction, but also improves their mechanical properties. The alloys exhibit a superior strength-ductility balance enabled by their unusual strain hardening behavior^1–4,6–11,13,15,17,18^. Furthermore, in contrast to ordinary malleable metals, where the strain hardening rate monotonically decreases during tensile deformation^19–24^, the Al-containing high-Mn steels (Fe–Mn-Al-C alloys) show a considerable recovery in the hardening rate with plastic deformation^2,4,6,7,9,11,16–18^. Such a strain hardening recovery or secondary strain hardening can significantly improve the ultimate tensile strength and ductility by delaying the necking point up to a higher strain level^22,24^.

Previous studies on the strain hardening recovery in polycrystalline materials attributed the recovery to their characteristic planar slip behavior. Yang et al.^25^ reported that strain hardening recovery occurs in polycrystalline Ni-Si alloys with large grain sizes. Via detailed microstructural characterizations, they showed that the planar dislocation slip leads to sequential activation of the secondary slip systems during tensile deformation. Thus, a sharp decrease in the dislocation mean free path (L) during the activation of the secondary slip systems is the primary reason for the strain hardening recovery. Recently, the slip activity-based strain hardening (SASH) model^26^ was proposed, considering the strain-dependent orientation factor, which spans from the lower-bound iso-stress Sachs model to the upper-bound iso-strain Taylor model. The SASH model successfully predicts the strain hardening recovery in polycrystalline metals with the presence of shearable precipitates, supporting that strain hardening recovery is a result of the planar slip-induced sequential activation of the slip systems.

Therefore, the strain hardening recovery of Fe–Mn-Al-C alloys can also be attributed to their characteristic planar slip behavior. Earlier seminal investigations^1–4,6–18,27^ have demonstrated that the deformation of the Fe–Mn-Al-C alloy mainly occurs due to the pronounced planar slip, which is strongly correlated to the ordered second phase, namely, κ-carbide precipitates. Adding Al promotes the formation of a fine distribution of nanometer-sized L'1_2_ atomic ordering (short-range ordering (SRO) or long-range ordering (LRO)) with an (Fe, Mn)_3_AlC_x_ (x ≤ 1) stoichiometry via spinodal decomposition^5,12,28,29^. Further, the ordered regions evolve into κ-carbide precipitates during aging at 500–650 ℃. Since the ordered precipitates have a similar atomic structure as the disordered matrix, the κ-carbides can be considered as modified face-centered cubic (FCC) structures, where Al atoms substitute the Fe atoms at the corner sites of the austenite unit cell. Based on this structural similarity, the κ-carbides are coherent or semi-coherent with the disordered austenite matrix, possessing a lattice misfit below 2%^15,16,18^. Although the exact origin for this planar glide is disputed^1,3,6,7,9,11,14–16,18^, a consensus has been reached that the "glide plane softening" phenomenon induces the planar glide^2,4^. In glide plane softening, the shearing of the resistive second phase (or precipitates) by leading dislocations enables the succeeding dislocations to easily glide on the same plane.

Thus, it is conceivable that the strain hardening recovery of Fe–Mn-Al-C alloys is inevitably dependent on the κ-carbide precipitation state. However, an understanding of the relation between the κ-carbide precipitates and the dislocation gliding behavior in the alloys is not yet firmly established. Furthermore, a clear interpretation of the strain hardening recovery mechanism based on dislocation plasticity is still missing. To address these gaps, the present investigation analyzes the effect of κ-carbide precipitates on the dislocation glide via in situ transmission electron microscopy (TEM), focusing on the interactions between the precipitates and gliding dislocations. Further, an attempt is made to understand the role of microstructural factors in the strain hardening behavior of Al-containing high-Mn lightweight steels using the experimental results.

## Results

### Precipitation state

**Figure 1:**
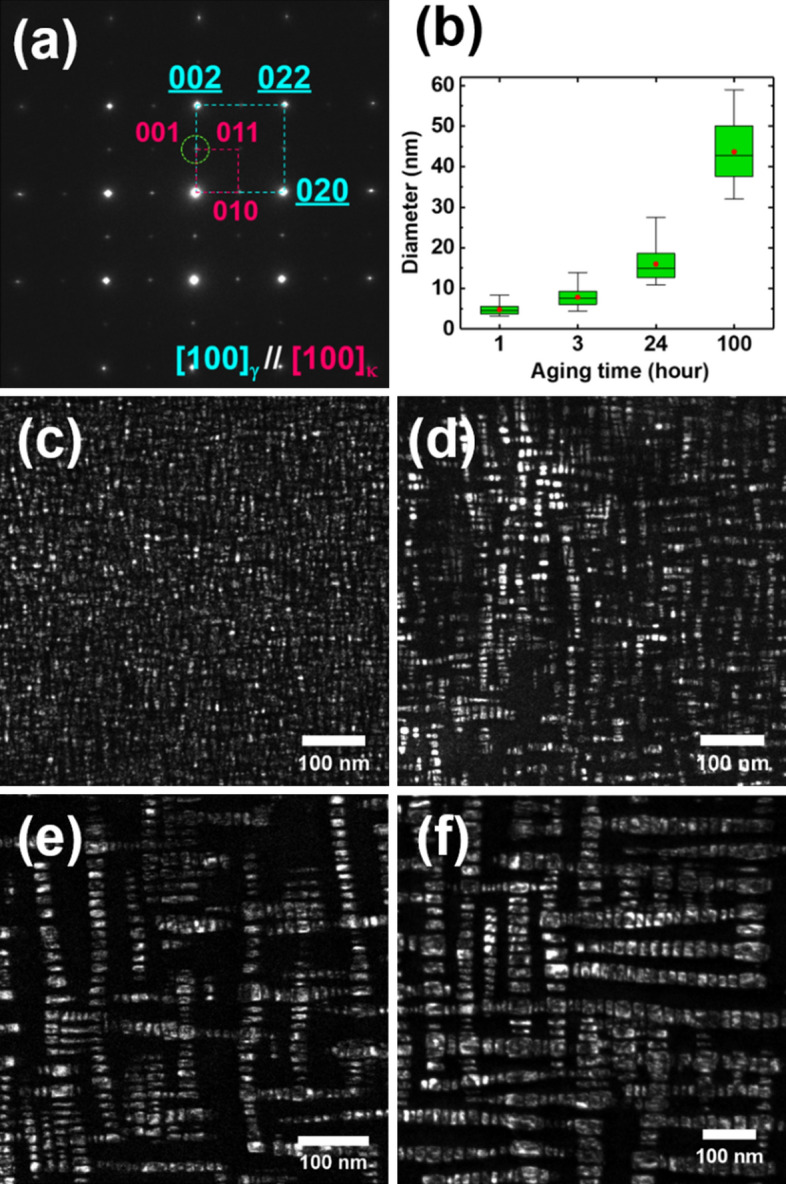
(**a**) Selected area diffraction pattern (SADP) of the aged Fe–Mn-Al-C alloy. (**b**) Size measurements of the κ-carbide with aging time. (**c**–**f**) Dark-field (DF) TEM images of 1, 3, 24, and 100 h-aged alloys, respectively.

**Figure 2:**
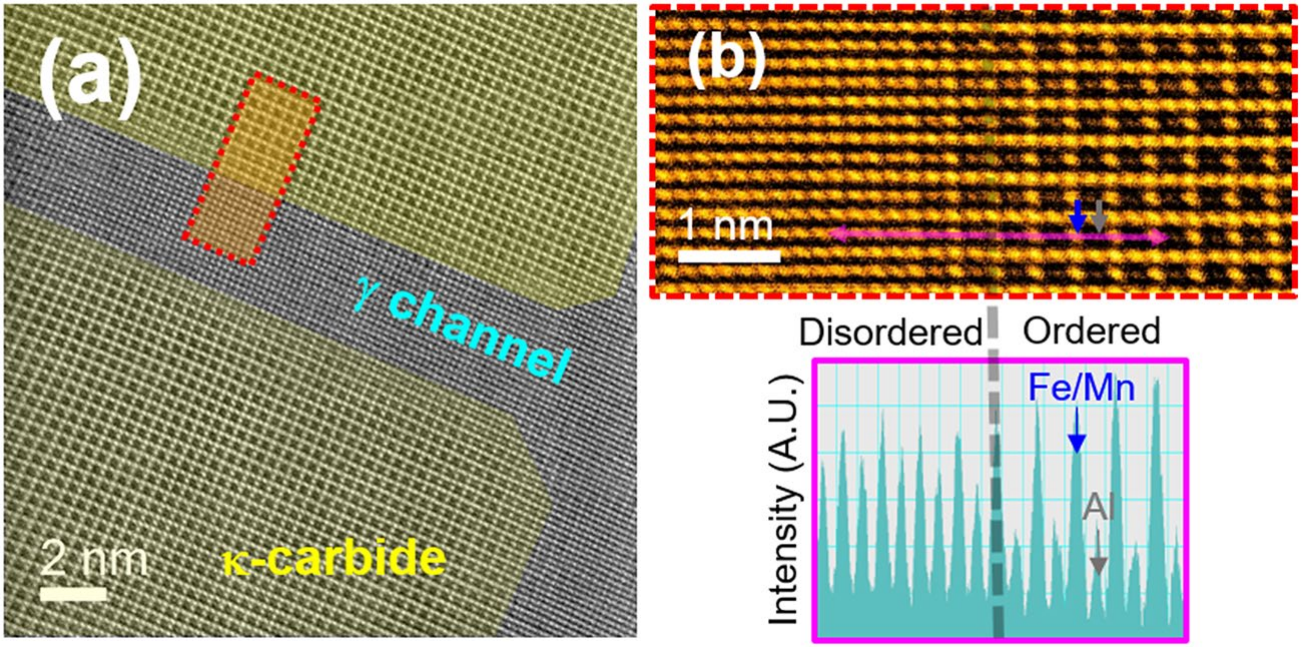
(**a**) High-angle annular dark-field (HAADF) STEM images of the 24 h-aged alloy acquired along the < 001 > direction. (**b**-upper) Magnified HAADF-STEM image of the coherent interface between the precipitate and austenite matrix. (**b**-lower) Intensity profile measured from the magenta line on the (**b**-upper) image.

**Figure 3:**
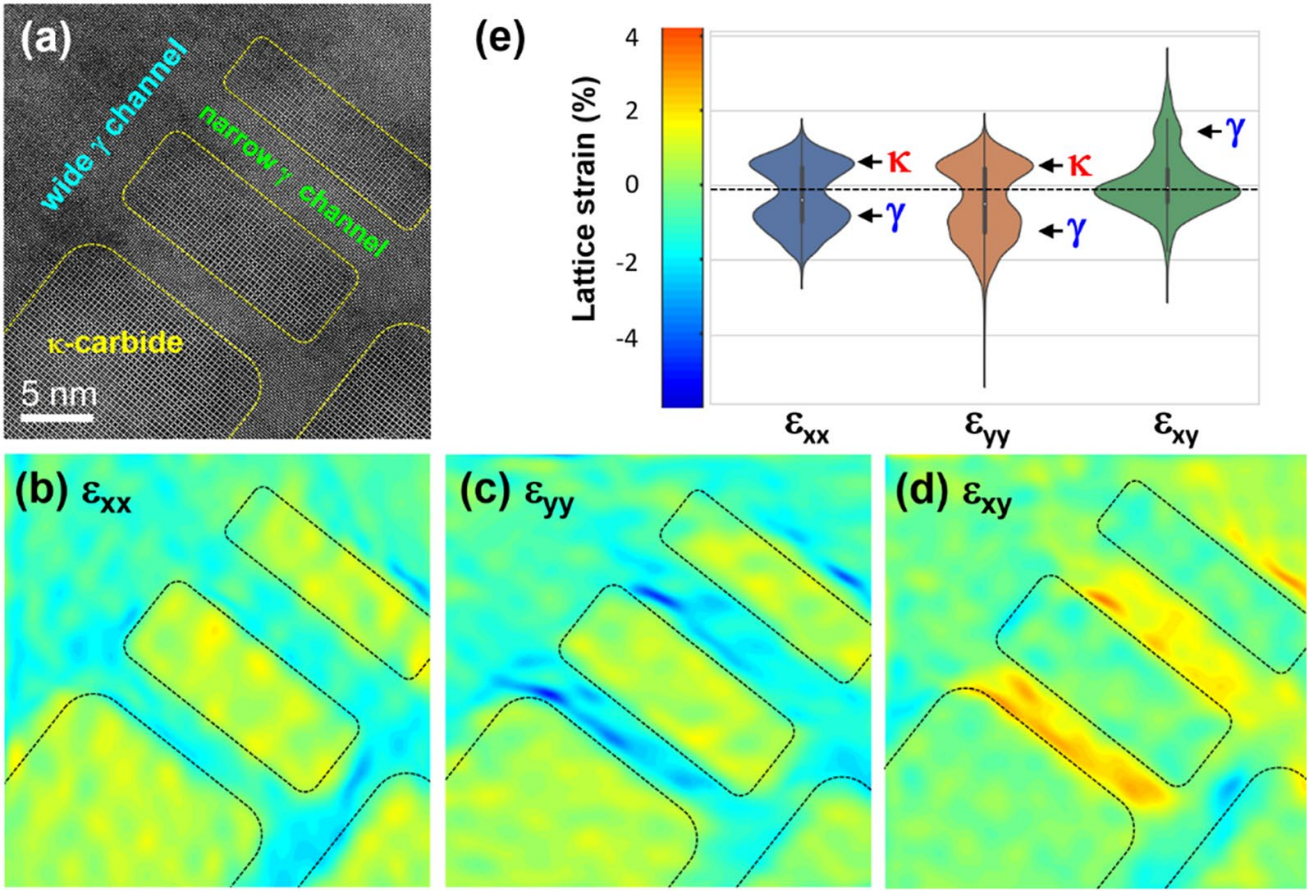
(**a**) HAADF-STEM image of the 24 h-aged Fe–Mn-Al-C alloy. (**b**–**d**) Geometric phase analysis (GPA) of (**a**). (**e**) Distribution of the lattice strains measured by GPA. The lattice strains were measured by using the < 100 > reflections of the austenite matrix.

Figure 1 shows the representative precipitation states of the Fe–Mn-Al-C alloys after isothermal aging at 600 °C for various durations. The selected area diffraction patterns (SADPs, Fig. 1a) taken from the 24 h-aged sample reveal that the κ-carbides have a cube-cube orientation relationship (OR) with the austenite matrix. Figure 1c–f shows the dark-field (DF) TEM images of the κ-carbides, which were acquired using the (001) superlattice spot in the SADP (Fig. 1a). The DF TEM images (Fig. 1c–f) show that the morphology of the κ-carbides is rectangular with {001} habit planes, similar to that of γ/γ’ (Ni_3_Al) in Ni-base superalloys^30–33^. The development of such a unique morphology is attributed to the preferential growth of the coherent {001} κ-_carbide_//{001}_austenite_ interface to minimize the surface energy. The mean diameters of the precipitates in the 1, 3, 24, and 100 h-aged samples were measured as 5, 8, 16, and 44 nm, respectively (Fig. 1b). As the aging time increases, the arrangement of κ-carbides changes from a random dispersion (Fig. 1c) to aligned stacks (Fig. 1d–f), i.e., the austenite matrix regions between the κ-carbides are categorized into narrow and wide channels. Previous atom probe tomography (APT) analysis^16,18,27^ has reported that most κ-carbides were grown into rectangular parallelepipeds rather than a cuboidal morphology, following which the carbide plates were aligned into "particle stacks" along the orthogonal < 001 > direction. High-angle annular dark-field (HAADF) STEM images of the 24 h-aged alloy acquired along a < 001 > direction (Fig. 2) visualized the coherent interface between the precipitate and austenite matrix. Further, the κ-carbides could be distinguished by the Al columns from the disordered matrix (Fig. 2b) because the light elements reduce the intensity of the HAADF-STEM image (Z-contrast) ^34–36^. Geometric phase analysis (GPA)^37,38^ of the 24 h-aged Fe–Mn-Al-C alloy (Fig. 3) was conducted to estimate the misfit strain in the vicinity of the κ-carbides. The ε_xx_ (Fig. 3b) and ε_yy_ (Fig. 3c) maps show that compressive strains (< 3%) are imposed on the narrow γ channels, whereas tensile strains (< 2%) are exerted on the κ-carbides. Accordingly, shear strains (ε_xy_) are intensively developed between the narrow γ channels (Fig. 3d). Nevertheless, it appears that the mean values of the strains are not large enough to generate misfit dislocations, revealing that the carbides are fully coherent with the matrix.

### Mechanical properties

**Figure 4:**
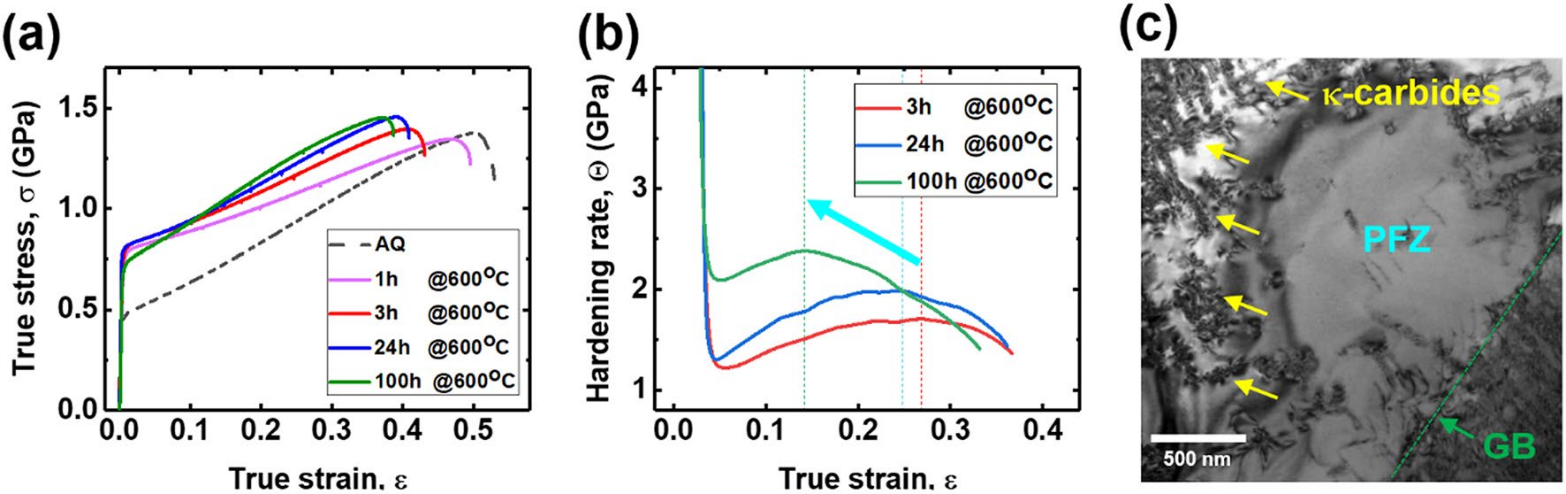
(**a**) True stress–strain curves of the aged alloys, and (**b**) their corresponding strain hardening rate curves measured at 293 °C. (**c**) BF-TEM image acquired in the vicinity of a grain boundary.

Figure 4a,b show the true stress–strain curves of the aged alloys and corresponding strain hardening rate curves measured at 293 °C respectively. Even after aging at 600 °C for 3 h, the yield strength (YS) of the alloys significantly increased (71%, 780 MPa) compared to that of the as-solution (AS) alloy (455 MPa). This high level of YS was maintained up to 24 h aging (800 MPa), and decreased to 706 MPa after 100 h. The decrease in YS after 100 h of aging can be attributed to the existence of a precipitate-free zone (PFZ) near the grain boundaries^8,39,40^, (Fig. 4c). Although the precipitates harden the matrix of the 100 h alloy, the relatively soft PFZ enables the initiation of the plastic deformation, leading to a reduction in YS. Furthermore, the maximum elongation is decreased as the aging time increases (43, 41, and 39% after 3 h, 24 h, and 100 h, respectively). The strain hardening rate curves of the alloys (Fig. 4b) exhibit humps, revealing that the strain hardening "recovery" occurs after a sharp drop at the yield point. As the aging time increases, the peak values of the humps increase, whereas the corresponding strain values decrease (cyan arrow in Fig. 4b). The strain hardening rate recovery is rapidly induced, but saturates early with increasing aging time. To understand this strain hardening recovery in terms of dislocation dynamics, we examined dislocation motions in the precipitation-hardened alloys by in situ tensile TEM experiments focusing on the interactions between the gliding dislocations and precipitates.

### In situ TEM results

**Figure 5:**
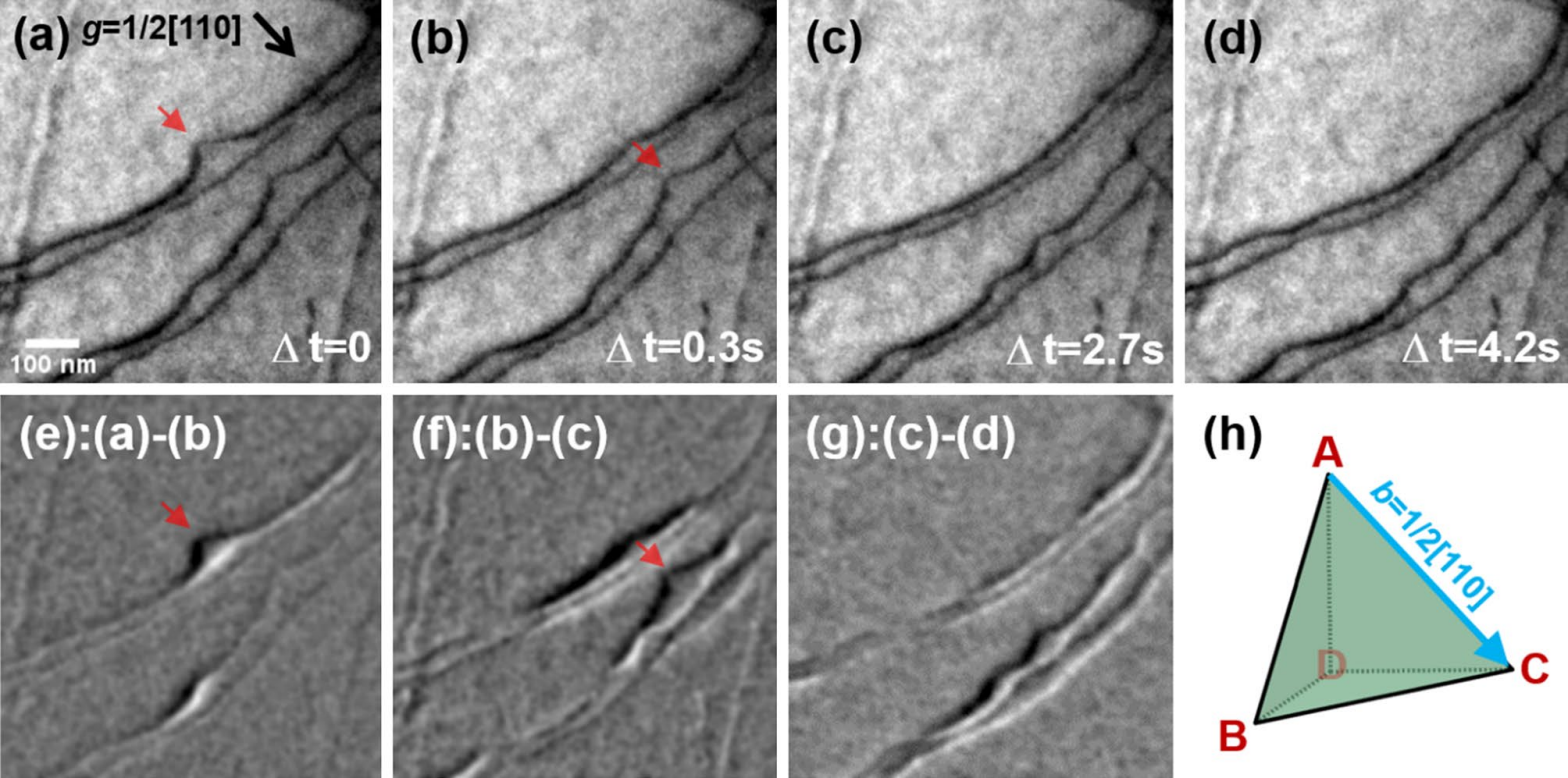
Dislocation gliding behavior in the 3 h-aged alloy. (**a**–**d**) Snapshots (BF-TEM images) taken from Supplementary Movie 1. (**e**–**g**) Differences between snapshots in (**a**–**d**). (**h**) Thompson's tetrahedron showing the sample geometry.

Figure 5 shows the typical consecutive motion of the dislocation glide in the 3 h-aged alloy. For a clearer visualization of the dislocation behavior, see Supplementary Movie 1. The sample geometry during the acquisition is provided in the form of Thompson’s tetrahedron (Fig. 5h), visualizing the {111} slip planes of FCC structure. The carbides exhibited a mottled feature on the matrix because the size of the κ-carbides was relatively small and the viewing direction was not parallel to the <001> direction. During deformation, pairs of dislocations with the Burgers vector (**b**) of 1/2[110] continuously glided on a {111} slip plane of the austenite matrix by shearing the κ-carbides precipitates. According to the ***g·b*** invisible criteria, the gliding dislocations were verified as mixed-type dislocations. The continuous flow of the planar gliding dislocations was comparable to that found during the deformation of an AS alloy^41^. However, the frequent impingement of the dislocation line (red arrows, Fig. 5) indicated that the gliding dislocations shear (cut) the carbides. The pairing of the gliding dislocations also confirmed that particle cutting is the dominant strengthening mechanism in the 3 h-aged alloy, because the pairing removes the anti-phase boundary (APB) in the sheared κ-carbides^14,15,18^.

**Figure 6:**
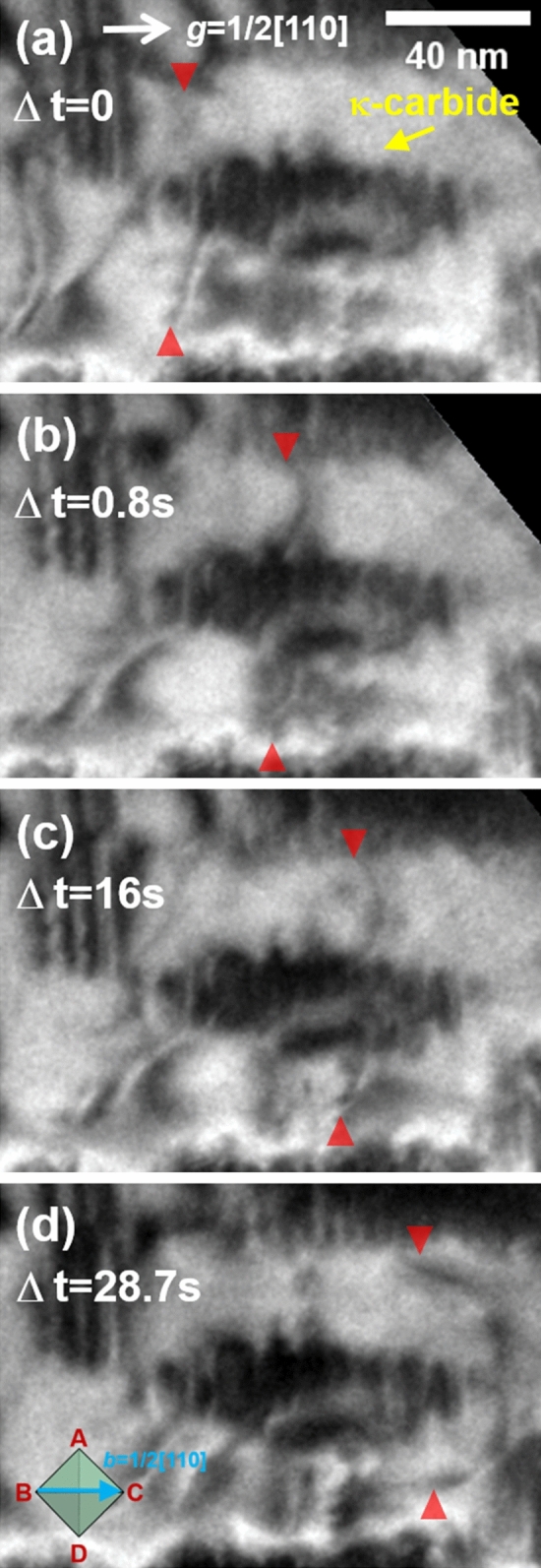
Dislocation-precipitate interaction during the deformation of the 24 h-aged alloy. (**a**–**d**) snapshots (BF-TEM images) taken from Supplementary Movie 2.

Figure 6 shows a representative dislocation-precipitate interaction during the deformation of the 24 h-aged alloy (detailed information on the dislocation movement is provided in Supplementary Movie 2). Similar to the 3 h-aged alloy, mixed-type dislocations with a Burgers vector of 1/2[110] were gliding on a {111} slip plane of the austenite matrix. However, in the 24 h-aged alloy, the κ-carbide blocks were clearly distinguishable from the carbide-free wide matrix channels (Section "Deformation mechanism"). Deformation proceeded by the irregular flow of gliding dislocations overcoming the hindrance of the κ-carbide blocks, contrary to the steady motion observed in the 3 h-annealed alloy. It is conceivable that the degree of strengthening by the precipitates increases as their size increases. The dislocations pass through the precipitate blocks without leaving any dislocation loop around the blocks, which implies that particle cutting is also the dominant deformation mechanism in the 24 h-aged alloy.

**Figure 7:**
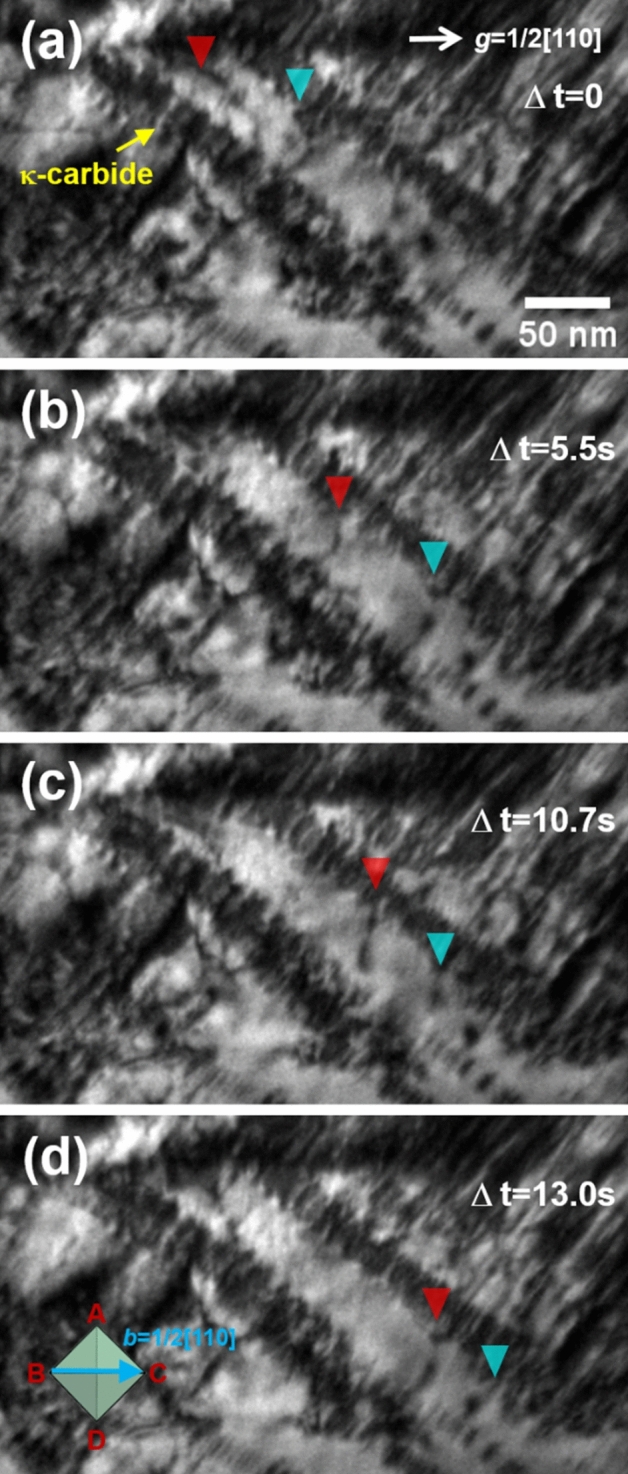
Dislocation-precipitates interaction in the 100 h-aged alloy. (**a-d**) snapshots (BF-TEM images) taken from Supplementary Movie 3. Cyan and red triangle marks denote the location of gliding dislocations in each of the sequential snapshots, revealing the dislocation’s gliding trajectory. Burgers vector of the gliding dislocations are indicated in the Thompson’s tetrahedron (lower left in (**d**)).

**Figure 8:**
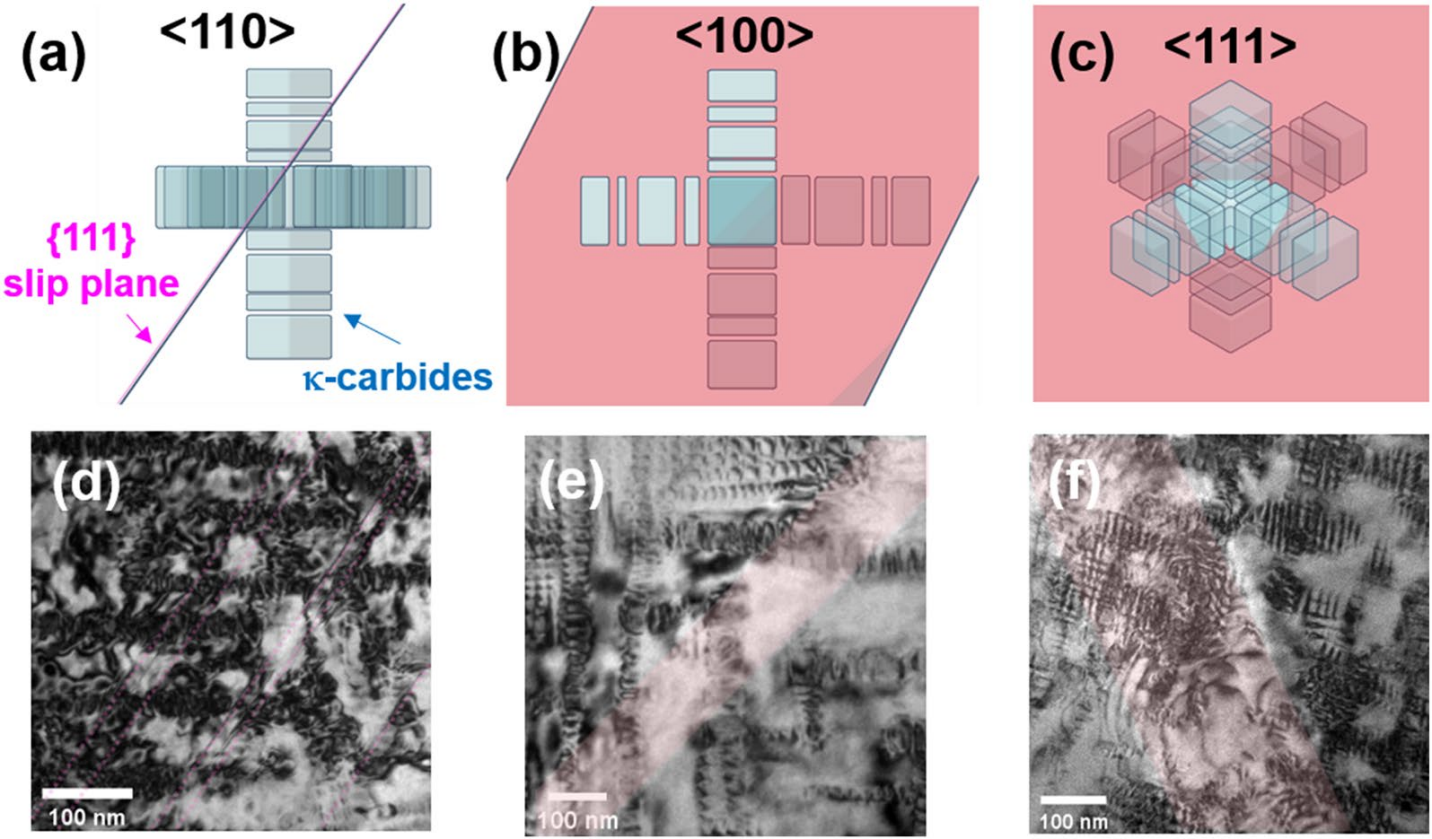
Precipitate-dislocation interactions observed along the < 110 > , < 100 > , and < 111 > directions. (**a**–**c**) Schematic illustrations of the κ-carbides blocks along with a {111} slip plane. (**d**–**f**) Bright-field (BF) TEM images of the 100 h-aged alloys from the viewing directions of < 110 > , < 100 > , and < 111 > . Gliding plane of dislocations in the FCC structure are visualized by shaded magenta area.

**Figure 9:**
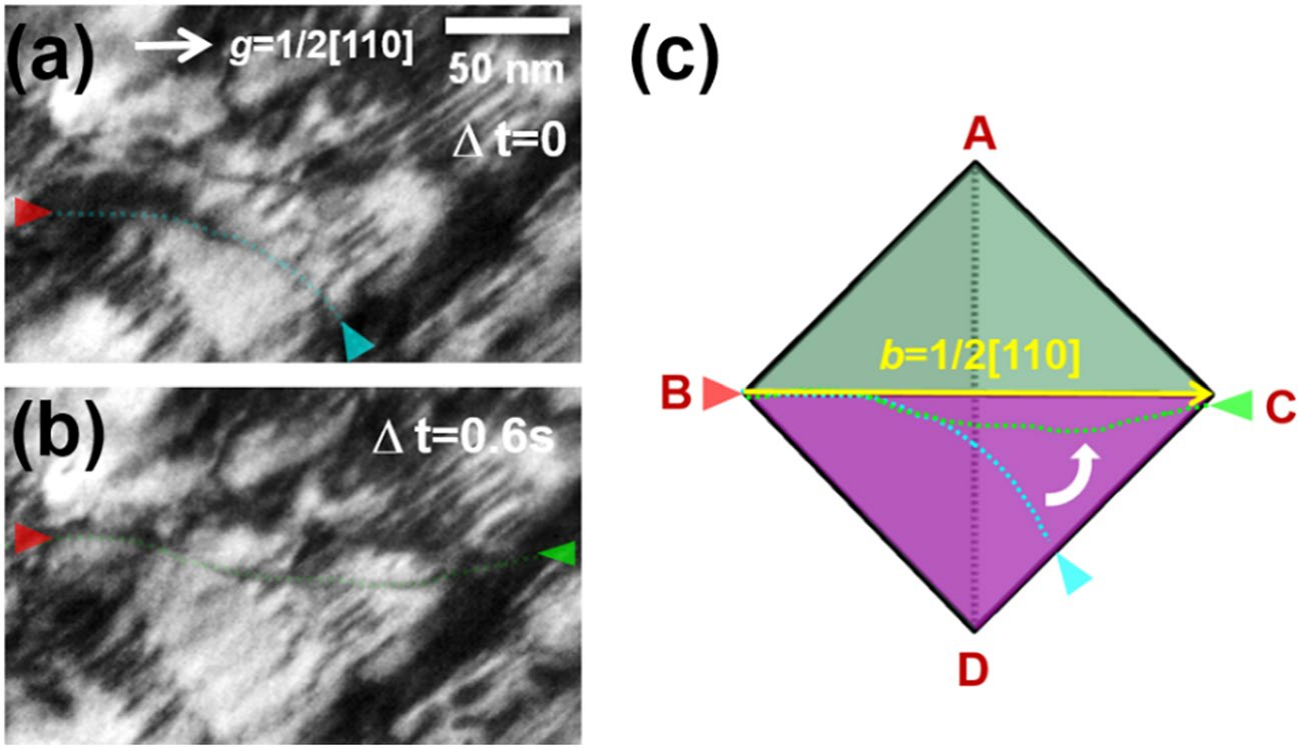
Bending of the dislocation by the precipitate pinning. (**a**,**b**) Snapshots (BF-TEM images) taken from Supplementary Movie 7. In the 100 h-aged alloy, (**a**) some gliding dislocations are (**b**) considerably bent up to 90°. The dislocation bending is illustrated in (**c**). The dislocation line vector of such a bent dislocation section is parallel to the dislocation gliding direction (i.e., Burgers vector, **b**).

**Figure 10:**
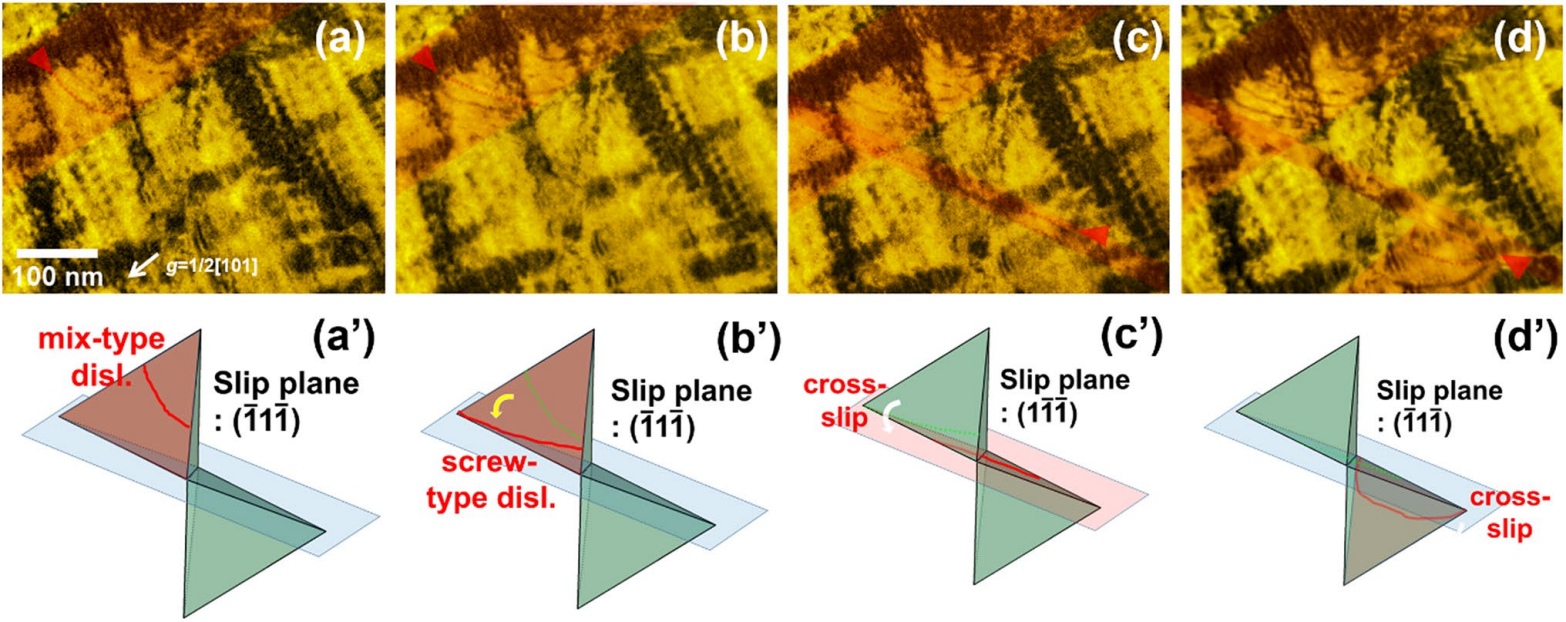
Cross-slips of the screw dislocations in the 100 h-aged alloy. (**a**–**d**) Snapshots (BF-TEM images) taken from Supplementary Movie 8, and (**a’**–**d’**) corresponding illustrations.

Figure 7 shows the dislocation-precipitate interaction in the 100 h-aged alloy (detailed information on the dislocation movement is provided in Supplementary Movie 3). In this case, the volume fraction of the κ-carbides is much higher than that of the 24 h-aged alloy, and the dislocation movements are further disturbed when gliding on the slip plane. The particle cutting by gliding dislocations is also clearly captured in the visuals of the 100 h-aged alloy (Fig. 8 and Supplementary Movie 4–6), where the particle-dislocation interactions were acquired from viewing directions of < 110 > , < 100 > , and < 111 > . When viewed from the < 110 > direction, it is particularly apparent that the precipitates were sheared by fine lines, implying that the dislocations shear the precipitates on particular glide planes (i.e., planar dislocation glide). As mentioned earlier, the shearing dislocations are readily bent by the pinning of the precipitates; thus, the dislocation line bending is more pronounced when the size of the precipitates is increased, as in the 100 h-aged alloy. Therefore, in the 100 h-aged alloy, some gliding dislocations are considerably bent up to 90° (Fig. 9 and Supplementary Movie 7). Thus, the dislocation line vector of such a bent dislocation section is parallel to the dislocation gliding direction (i.e., Burgers vector, ***b***), implying that the bent section of the dislocations is transferred to the screw-type dislocation. It is known that screw-type dislocations in FCC materials easily transit their slip plane because their Burgers and line vectors are not confined in a unique plane^20,24,42,43^. Therefore, the screw-type dislocations originating from dislocation bending are easy to cross-slip onto other slip systems. Figure 10 shows such cross-slips of the screw dislocations in the 100 h-aged alloy (from Supplementary Movie 8). In addition, it is noteworthy that the cross-slip consequently leads to the activation of the secondary slip system.

## Discussion

### Deformation mechanism

The deformation mechanism in precipitation-hardened materials can be determined by comparing the YS increment (strengthening) using the Orowan looping (∆σ_Orowan_) and particle shearing (∆σ_shearing_) mechanisms, in which the deformation mechanism with a lower ∆σ dominates^5,14,24,44^.

Strengthening by the Orowan looping mechanism is expressed as^5,14,24,44^,

$$\Delta {\sigma }_{Orowan}=M\frac{0.4Gb}{\pi \sqrt{1-\upsilon }}\frac{\mathrm{l}\mathrm{n}(2\stackrel{-}{r}/b)}{2\stackrel{-}{r}(\sqrt{\frac{\pi }{4f}}-1)}$$

where *M*, *G*, *b*, and *v* are constants (mean orientation factor for FCC polycrystalline matrix, shear modulus of austenite matrix, the magnitude of the Burgers vector of 1/2[110], and Poisson’s ratio, respectively). The equation consists of two variables, the radius ($r=\frac{\stackrel{-}{\mathrm{r}}}{\sqrt{2/3}}$) and volume fraction (*f*) of the precipitates, and ∆σ_Orowan_ increases as the *f* of the precipitates increases. However, at a fixed *f*, ∆σ_Orowan_ is inversely proportional to the size of the precipitates. Hence, the Orowan looping mechanism is likely to operate in overaged precipitation-hardened alloys^22,24,43–45^.

Furthermore, there are three major factors that contribute to the strengthening caused by the particle shearing mechanism (∆σ_shearing_): coherency strengthening (∆σ_coherency_), modulus mismatch strengthening (∆σ_ms_), and order strengthening (∆σ_order_)^5,14,24,44^. Among them, coherency strengthening, the most determinant strengthening factor for particle shearing mechanism, is estimated as follows^5,14,24,44^:

$$\Delta {\sigma }_{coherency}=KM{\left(G{\varepsilon }_{c}\right)}^{\frac{3}{2}}{(\frac{rf}{0.5Gb})}^{\frac{1}{2}}$$

where *K* is a constant and *ε*_*c*_ is the lattice misfit strain. Contrary to ∆σ_Orowan_, the growth of the precipitates increases ∆σ_coherency_, which inhibits the shearing of the precipitates by dislocations. On the contrary, in the case of aged Fe–Mn-Al-C alloys, the fully coherent κ-carbide precipitates effectively reduce the lattice misfit strain (*ε*_*c*_≈0), causing ∆σ_coherency_ to be negligible, and facilitating the shearing of the precipitates. As confirmed by GPA (Fig. 3), the precipitation of κ-carbides generates a negligible amount of lattice strains (< 3%); consequently, particle shearing becomes the primary deformation mechanism even with relatively large-sized κ carbide precipitates. Our in situ TEM results also confirmed that the deformation of the aged Fe–Mn-Al-C alloys occurs via particle shearing. Detailed calculations and microscopic investigations in previous studies^16,18^ suggest that the particle shearing mechanism is energetically favorable when the precipitate size is below a critical value.

When particle shearing occurs, dislocation shearing reduces the effective size of the precipitates as follows^16,26,30,43^,

$${r}_{eff}=\,\stackrel{-}{r}-\frac{nb}{2}$$

where n is the number of shearing dislocations. In other words, each shearing reduces the effective radius of the precipitates, which helps the following dislocations shear the precipitates on the same slip plane (known as “glide plane softening”)^2,3,6,7,9,11,14–16,18^. Therefore, the particle shearing in the aged Fe–Mn-Al-C alloys leads to the planar dislocation glide. In summary, it can be concluded that the high degree of lattice coherency between the κ-carbides and austenite matrix enables the dislocations to shear the precipitates, reducing the resistivity of the precipitates toward the dislocation glide, and thereby causing a pronounced planar dislocation glide.

### Strain hardening behavior

Recently, the SASH model^26^ was proposed to quantitatively interpret the strain hardening recovery. It comprehensively interprets the strain hardening recovery behavior of twinning-induced plasticity (TWIP) steels and precipitation-hardened Ni alloys by considering the three dominating factors: forest dislocation density, orientation factor (M"), and dislocation mean free path (mobile dislocation density). The governing ordinary differential equations can be written as,

$$\frac{d{\rho }_{f}}{d{\epsilon }_{p}}=M{\text{''}}(\frac{1}{\boldsymbol{b}L}-{K}_{2}{\rho }_{f})$$

$$\frac{dM"}{d{\epsilon }_{p}}={K}_{M}({M}_{s}"-M")$$

$$\frac{dL}{d{\epsilon }_{p}}=-{K}_{L}(L-{L}_{s})$$

**Figure 11:**
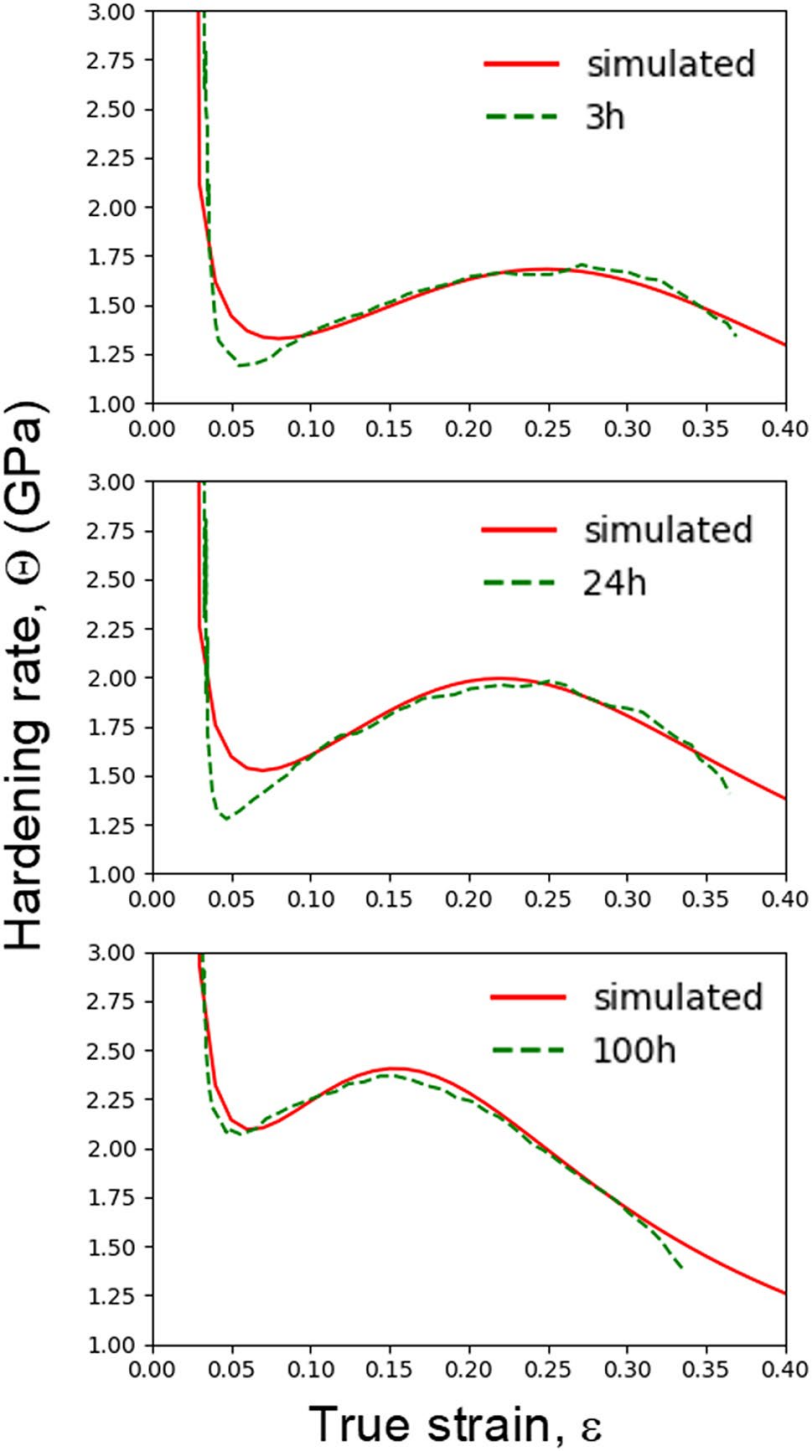
Strain hardening rate curves of the aged alloys (green dotted lines), and their corresponding simulated strain hardening fitted using the SASH model (red lines).

ρ_f_ is the forest dislocation density, ε_p_ is a plastic strain, and M_S_” and L_S_ are the saturation value of the orientation factor (M”) and the dislocation mean free path (*L*), respectively. K_M_ is related to the orientation factor, which is no longer constant and varies with deformation. The variable K_2_ is related to dislocation removal by dynamic recovery. K_L_ defines the rate at which the dislocation mean free path approaches its saturation value (L_S_). The strain hardening rate curves of the precipitation-hardened alloys were fitted using the SASH model (Fig. 11, Table 1 (fitting parameters)). It can be seen that the most remarkable feature is the change in K_L_ values with increasing aging time. The K_L_ values of the 3 h and 24 h-aged alloys are comparable, but that of the 100 h-aged alloy is quite large, implying that the dislocation mean free path in the 100 h alloy is more rapidly saturated than in the 3 h or 24 h-aged alloys. This trend in the mechanical property corresponds to the dislocation behaviors observed in the in situ experiment. As mentioned earlier, a frequent cross-slip of gliding dislocations was noted during the deformation of the 100 h-aged alloys. The bigger and harder κ-carbides act as effective barriers against dislocation motion and subsequently bend the dislocations by making the line vector (***u***) parallel to the Burgers vector (***b***); the type of dislocations is changed from edge to screw. The screw dislocations are easy to cross-slip, promoting early activation of the secondary slip system. Accordingly, the simultaneous activation of multiple slip systems leads to additional barriers to the dislocation glide, and consequently, an abrupt decline in the dislocation mean free path.

**Table 1:** Parameters for fitting the strain hardening rate curves of the precipitation-hardened alloys to the SASH model.

Alloy	3 h	24 h	100 h	Description
ρ_0_	1*10^11^	1*10^11^	1*10^11^	Initial value of dislocation density^26^
M_0_	2.2	2.2	2.2	Initial value of M^26^
M_S_	3.0	3.0	3.0	Saturated value of M^26^
L_0_ (m)	3*10^–6^	3*10^6^	3*10^6^	Initial value of L
L_S_ (m)	1*10^7^	1*10^7^	1*10^7^	Saturated value of L
K_M_	10	10	10	Rate of M approaching M_S_
K_L_	13	15	20	Rate of L approaching L_S_
K_2_	0.2	0.3	0.5	Rate of dislocation recovery

**Figure 12:**
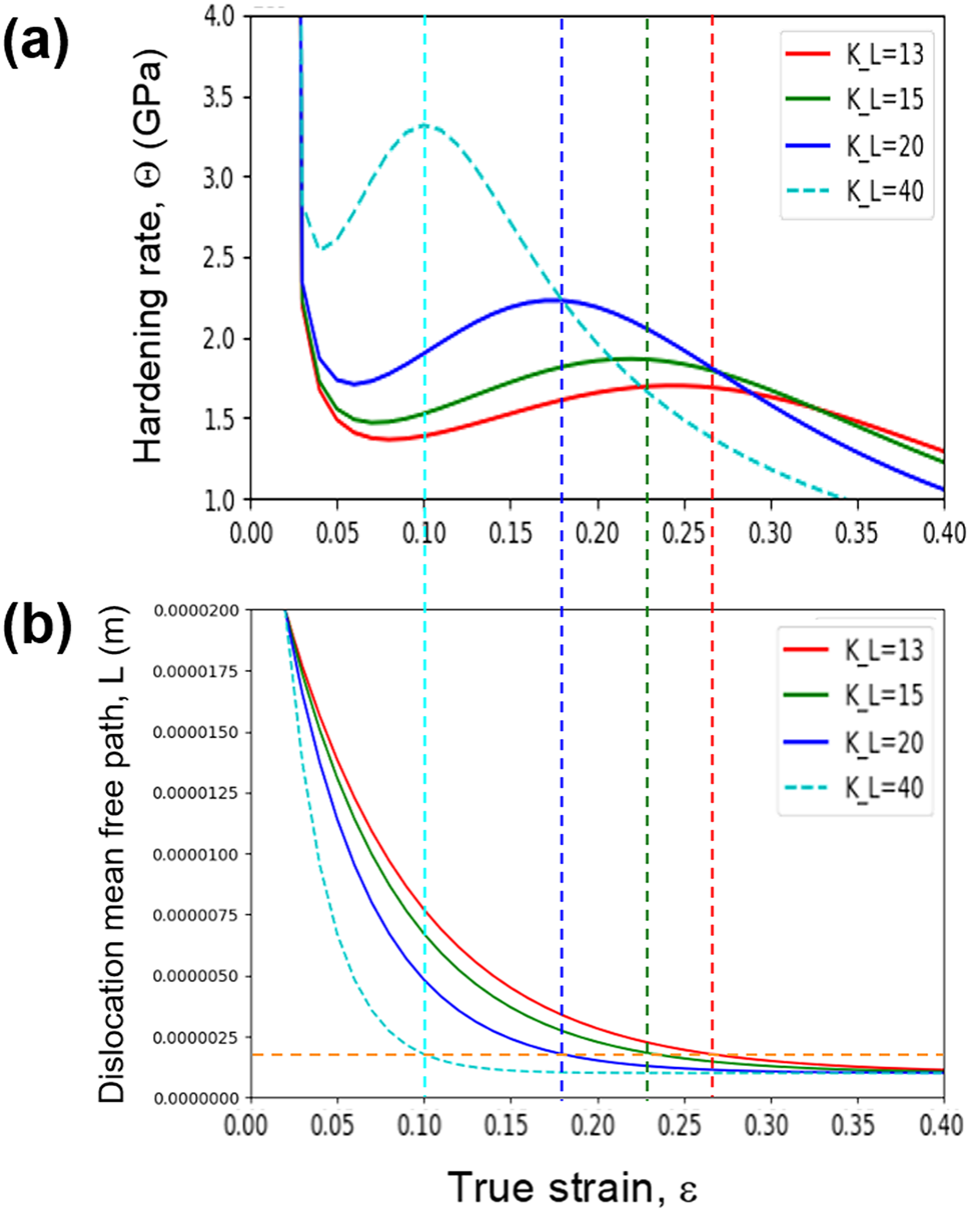
Strain hardening curves simulated by the SASH model (upper), and their corresponding dislocation mean free path graph (lower).

Figure 12 shows the simulated strain hardening curves according to the SASH model (Fig. 12a), and their corresponding dislocation mean free path graphs (Fig. 12b). To reveal the relationships between the strain hardening recovery and dislocation mean free path, fitting parameters for the 100 h-aged alloy from Table 1 were used to simulate the strain hardening rate curves. The strain hardening recovery activated when the dislocation mean free path was larger than a critical value (~ 1.7 µm). It is reasonable to deduce that the early disappearance of the strain hardening recovery in the 100 h-aged alloy is due to the rapid decrease in the dislocation mean free path. Hence, preserving the dislocation mean free path by suppressing the cross-slip or promoting the planar glide might be essential to induce strain hardening recovery.

Assuming that the strain hardening rate recovery of the precipitation-hardened Fe–Mn-Al-C alloys can be enabled by the planar slip deformation, the strain hardening recovery behavior in the TWIP and phase transformation-induced plasticity (TRIP) steels also can be explained by their characteristic planar slip behaviors. Due to their low stacking fault energy, the dislocation behaviors in TWIP and TRIP steels are characterized by the pronounced planar glide of Shockley partial dislocations^46–51^, generating abundant stacking faults, which are the building blocks for twin and epsilon martensite^46–51^. Therefore, in TWIP and TRIP steels, cross-slip and the simultaneous activation of multiple slip systems are inhibited by the planar glide of the Shockley partial, allowing the recovery of the strain hardening rate.

Thus, if we could design a microstructure where the dislocations glide in a planar manner, and dislocation cross-slip and the simultaneous onset of multiple slip systems is suppressed with the ongoing plastic deformation, the strain hardening recovery could be utilized optimally to obtain enhanced mechanical properties.

## Conclusions

In the present study, we investigated the effect of κ-carbide precipitates on the strain hardening behavior of aged Fe–Mn-Al-C alloys from a microscopic point of view. Via comprehensive TEM analyses, including HAADF-STEM and in situ straining TEM, the following conclusions were drawn:

- Rectangular parallelepiped-shaped κ-carbides with coherent {001} κ-_carbide_//{001}_austenite_ habit planes were precipitated after the isothermal aging of Fe–Mn-Al-C alloys at 600 °C for various times. The sizes of the precipitates increased with the aging time, but the lattice coherency between the precipitates and matrix was preserved even after 100 h of aging.
- By the precipitation of κ-carbides, the YS and flow stress were considerably increased compared to those of the AS alloy. Recovery of the strain hardening rate occurred in the aged Fe–Mn-Al-C alloys. As the aging time increased, the strain hardening recovery saturated faster.
- Results of the in situ tensile TEM experiments confirmed that the particle shearing mechanism occurs in the aged Fe–Mn-Al-C alloys. The origin behind this mechanism was deduced to be the considerable reduction in ∆σ_shearing_ by the high degree of lattice coherency between the κ-carbides and austenite matrix.
- During deformation of the 100 h-aged alloy, some gliding dislocations were strongly pinned by the large κ-carbide blocks, and consequently, severely bent. The bent dislocations were easy to cross-slip into other slip planes, promoting the activation of multiple slip systems. The abrupt decline in the dislocation mean free path was attributed to the activation of multiple slip systems, leading to the saturation of the strain hardening rate recovery at lower strain levels.
- It can be assumed that the strain hardening recovery is closely related to the pronounced planar dislocation glide. Accordingly, if a microstructure where the dislocations glide in a planar manner is designed, the strain hardening recovery can be utilized to enhance the mechanical properties in lightweight steels.

## Methods

Methods for sample preparation and microstructure analysis are adopted from the author’s previous study^41^. The chemical composition of the investigated alloy was Fe-32Mn-8.9Al-0.78C (wt. %). The alloy was melted in an induction furnace and cast into a 60 kg ingot (180 mm dia. × 250 mm height) under vacuum. The ingot was homogenized for 5 h at 1220 °C and forged into a block (70 × 120 × 750 mm); subsequently, plate-shaped samples (70 × 120 × 20 mm) were cut from the block, followed by solution treatment at 1050 °C for 5 h and subsequent water quenching. The as-solution-treated samples were isothermally annealed at 600 °C for various times (1, 3, 24, and 100 h) and water-quenched to promote the formation of κ-carbides. Cylindrical tensile samples were obtained from the plate sample with a gage dimension of 6.25 mm diameter and 25 mm length. Tensile tests were carried out at 293 °C with a strain rate of 0.008/s using a tensile test machine (INSTRON 5982, Canton, MA). The precipitation state of the annealed samples was investigated using TEM. For a conventional post-mortem TEM analysis, disks with a diameter of 3 mm were mechanically polished to approximately 100 μm thickness and electrochemically etched by an electrolytic twin-jet polishing machine (TenuPol-5, Struers). The electrochemical etching was conducted at 10 V and 70 mA with a mixed solution of 10% perchloric acid and 90% methanol at − 20 °C. Samples for the in situ tensile TEM experiments were prepared to specific dimensions (3 mm × 12 mm) by punching the polished foil using a custom-made foil puncher. The in situ TEM samples were also finally etched using the aforementioned etching condition. The TEM (JEM-2100F, JEOL Ltd, Japan.) equipment was operated at an acceleration voltage of 200 kV, and an in situ straining TEM holder (straining in situ holder- model 654, Gatan, Inc, USA.) was used for the in situ experiments. Average size of the carbide is calculated by using the particle analyzing algorithm in ImageJ software. To measure the average size of the κ-carbides, we used 10 DF-TEM images from each sample.

## Supplementary Information

Supplementary Information 1.

Supplementary Video 1.

Supplementary Video 2.

Supplementary Video 3.

Supplementary Video 4.

Supplementary Video 5.

Supplementary Video 6.

Supplementary Video 7.

Supplementary Video 8.
