# Supplementary material for: Strain hardening recovery mediated by coherent precipitates in lightweight steel
Source: Sci Rep. 2021 Jul 14;11:14468. doi: 10.1038/s41598-021-93795-4 (PMC8280213; doi:10.1038/s41598-021-93795-4)
Supplement: Supplementary file 1 — Supplementary Information 1. [file 41598_2021_93795_MOESM1_ESM.pdf]

Strain hardening recovery mediated by coherent precipitates in lightweight steel

Sung-Dae Kim[a], Seong-Jun Park[a], Jae hoon Jang[a], Joonoh Moon[a], Heon-Young Ha[a], Chang-Hoon Lee[a], Hyungkwon Park[a], Jong-Ho Shin[b] and Tae-Ho Lee[a,*]

Supplementary Movie 1. In-situ TEM video of the dislocation's typical consecutive glides during tensile deformation in the 3 h-aged alloy (double-speeded, frame rate of 60fps).

Supplementary Movie 2. In-situ TEM video of the dislocation-precipitate interaction during the deformation in the 24 h-aged alloy (double-speeded, frame rate of 60fps).

Supplementary Movie 3. In-situ TEM video of the dislocation-precipitate interaction during the deformation in the 100 h-aged alloy (double-speeded, frame rate of 60fps).

Supplementary Movie 4. In-situ TEM video of particle cutting by gliding dislocations in the 100 h-aged alloy, acquired from viewing directions of <110> (double-speeded, frame rate of 60fps).

Supplementary Movie 5. In-situ TEM video of particle cutting by gliding dislocations in the 100 h-aged alloy, acquired from viewing directions of <100> (double-speeded, frame rate of 60fps).

Supplementary Movie 6. In-situ TEM video of particle cutting by gliding dislocations in the 100 h-aged alloy, acquired from viewing directions of <111> (double-speeded,

frame rate of 60fps).

Supplementary Movie 7. In-situ TEM video of the dislocation-precipitate interaction during the deformation in the 100 h-aged alloy, showing some gliding dislocations are considerably bent up to 90° (double-speeded, frame rate of 60fps).

Supplementary Movie 8. In-situ TEM video of the dislocation-precipitate interaction during the deformation in the 100 h-aged alloy, showing ross-slips of the screw dislocations in the 100 h-aged alloy (double-speeded, frame rate of 60fps).
